# Blood Glucose Level Forecasting on Type-1-Diabetes Subjects during Physical Activity: A Comparative Analysis of Different Learning Techniques

**DOI:** 10.3390/bioengineering8060072

**Published:** 2021-05-26

**Authors:** Benedetta De Paoli, Federico D’Antoni, Mario Merone, Silvia Pieralice, Vincenzo Piemonte, Paolo Pozzilli

**Affiliations:** 1 Unit of Computer Systems and Bioinformatics, Department of Engineering, Università Campus Bio-Medico di Roma, 00128 Rome, Italy; benedetta.depaoli@alcampus.it (B.D.P.); f.dantoni@unicampus.it (F.D.); 2 Unit of Diabetology and Endocrinology, Department of Medicine, Università Campus Bio-Medico di Roma, 00128 Rome, Italy; s.pieralice@unicampus.it (S.P.); p.pozzilli@unicampus.it (P.P.); 3 Unit of Chemical Engineering, Department of Engineering, Università Campus Bio-Medico di Roma, 00128 Rome, Italy; v.piemonte@unicampus.it

**Keywords:** diabetes, time series forecasting, online learning, physical activity, precision medicine, neural network

## Abstract

Background: Type 1 Diabetes Mellitus (T1DM) is a widespread chronic disease in industrialized countries. Preventing blood glucose levels from exceeding the euglycaemic range would reduce the incidence of diabetes-related complications and improve the quality of life of subjects with T1DM. As a consequence, in the last decade, many Machine Learning algorithms aiming to forecast future blood glucose levels have been proposed. Despite the excellent performance they obtained, the prediction of abrupt changes in blood glucose values produced during physical activity (PA) is still one of the main challenges. Methods: A Jump Neural Network was developed in order to overcome the issue of predicting blood glucose values during PA. Three learning configurations were developed and tested: offline training, online training, and online training with reinforcement. All configurations were tested on six subjects suffering from T1DM that held regular PA (three aerobic and three anaerobic) and exploited Continuous Glucose Monitoring (CGM). Results: The forecasting performance was evaluated in terms of the Root-Mean-Squared-Error (RMSE), according to a paradigm of Precision Medicine. Conclusions: The online learning configurations performed better than the offline configuration in total days but not on the only CGM associated with the PA; thus, the results do not justify the increased computational burden because the improvement was not significant.

## 1. Introduction

Type 1 Diabetes Mellitus (T1DM) is an autoimmune condition characterized by dysfunctions in insulin secretion from the islets of Langerhans in the pancreas. The main consequence of T1DM is the increase of the glucose concentration in blood. In order to reduce the incidence of diabetes-related complications and improve the quality of life of people with diabetes, it is essential to prevent blood glucose levels from going from the normal physiological range (70–180 mg/dL) to values below 70 mg/dL (hypoglycaemia) or above 180 mg/dL (hyperglycaemia) [1]. Modern therapies for the management of T1DM involve the use of Continuous Glucose Monitoring (CGM). Such devices exploit micro-invasive subcutaneous glucose sensors to track the blood glucose levels for 24 h a day.

Although regular physical activity is important for people living with T1DM for a variety of health reasons [2], most of them are unable to manage the consequences because the intensity [3], duration, and type of physical activity have a significant impact on glucose homeostasis [4,5], which can lead to potential episodes of hypoglycaemia or hyperglycaemia. The former is mainly observed during aerobic physical activity, whereas the latter is observed during anaerobic exercise.

In recent years, efforts have been made toward the development of an artificial pancreas [6], i.e., an automated therapeutic system intended to manage blood glucose levels and reduce the risks related to diabetes. Several predictive models have been developed that can identify the glycaemic trends of people with T1DM starting from the measurements detected by the CGM, most of which resort to artificial intelligence [7]. In particular, these models use Machine Learning algorithms that exploit the CGM structure and its temporal correlation, treating it with the analysis methodologies used for time series [8]. The methods present in the literature can be differentiated based on three main types of approaches:

1. Regression tasks, in which a prediction horizon is fixed and the point value of the CGM is predicted starting from known values of the features [9], or classification tasks, in which the model attempts to predict whether or not a hypo/hyperglycaemic event will occur within a fixed prediction horizon [10];
2. The univariate approach, in which the CGM is the only model input [11], or multivariate approach, in which CGM is considered in correlation with further features [12];
3. Precision Medicine, in which the model aims to adapt to patient-specific data [13], or k-Fold Cross Validation, which consists of testing one patient’s data after having trained the model on the data of the other patients in the dataset [14].

In addition, either offline or online training can be used for predictive models. The most common is offline training [11,13,15], in which the model is trained only once using a subset of the available data, and then tested on subsequent data without being updated. On the other hand, during online training, the update of the model takes place at each new sample or new batch of data: forecasts receive immediate feedback that is used to improve the accuracy of subsequent predictions.

Despite the excellent performance obtained by predictive models, the prediction of abrupt changes in blood glucose values produced during sports remains one of the main challenges in this sector. Physical activity has rarely been addressed in the literature, mainly due to the difficulties in data collection. In addition, the works addressing this task usually resort to multivariate approaches that ask the patient to manually supply data concerning their state or integrate their data from several heterogeneous sensors [16,17], or consider a dataset composed of virtual patients [18,19].

One of the most notable works was proposed by Hobbs et al. [17], who applied predictor-based subspace identification to a glucose prediction model. This multivariate model exploited the physiological variables and CGM data of 32 adolescents with T1DM collected in two ski and snowboard camps and achieved an average forecasting RMSE of 29 mg/dL. A different approach was pursued by Reddy et al. [20], who performed a classification task to predict hypoglycaemia at the start of exercise using a Decision Tree and a Random Forest model.

Both models were trained using a multivariate meta-dataset based on 154 observations of in-clinic aerobic exercise in 43 adults with T1DM, and then validated using a validation dataset with 90 exercise observations collected from 12 different adults with T1DM. The Decision Tree predicted hypoglycaemia with 79.6% accuracy, whereas the Random Forest achieved a 86.7% accuracy.

In principle, the online training should make a model able to dynamically adapt to new data patterns and have an advantage in predicting unexpected changes, such as those that occur during physical activity. A further benefit in the management of blood glucose levels forecasting during sports could be derived from Reinforcement Learning, consisting of algorithms that allow the training of an agent to take actions in a given environment in order to achieve preferable states [21]. In such a system, for every action taken, the agent receives positive or negative reinforcement from the environment. Reinforcement Learning techniques have been tested for glycaemic prediction and control [22], and their flexibility of adaptation has been highlighted.

In this work, we exploit the data of six adults suffering from T1DM who regularly perform physical activity to develop a predictive model capable of effectively forecasting future glucose levels during sports. The experimental work has seen the application of a Jump Neural Network [15] to perform a regression task with a univariate approach. This latter choice was made in order to reduce, as much as possible, the burden of the patient without requiring them to supply data manually or to wear unnecessary sensors.

A Precision Medicine approach was adopted, which, from the experimental results proved to be the most effective. With regard to the methods of application of the algorithm, three different configurations were developed, which include: a model with offline training, a model with online training, and a model with online training and a loss function that incorporates a negative reinforcement in cases with great differences between the predicted and real values. A comparative analysis was finally conducted, with the aim of determining whether the performance of a model with offline training could be exceeded by models with online updates, with or without penalty contribution.

## 2. Materials and Methods

The Division of Endocrinology and Diabetology of Campus Biomedico polyclinic of Rome supplied anonymized data of six subjects suffering from T1DM aged between 23 and 52 (average 39±10) that held regular physical activity and exploited CGM. The data were accompanied by information provided by the patients regarding the days and times in which physical activity was performed and its type. In detail, three of the participating subjects performed anaerobic activities (gym, sailing, and home workouts for patients 1, 2, and 3, respectively), whereas the remaining performed aerobic activities (paddle/bicycle, belly dance, and eight-a-side football for patients 4, 5, and 6, respectively).

For each patient, several sensor disconnections occurred during the monitoring period, which made it difficult for an artificial intelligence model to learn the patterns of a time series [23]. As a consequence, we decided to include in this study only those days for which the monitoring was continuous for 24 h. After excluding incomplete days, the amount of data collected ranged from a minimum of 6 days for patient 1 to a maximum of 81 for patient 4 (average 31±28) and a number of physical activity events ranging from a minimum of two for patient 2 to a maximum of eight for patient 4 (average 5±2).

With the purpose to make a prediction to 30 min, in the labelling process, the CGM value of the time stamp (t+30) was considered as a target for the training of the CGM sample at time stamp *t*. The three configurations developed in this work used a Jump Neural Network, which is a particular feed-forward neural network whose inputs are connected not only to the first hidden layer but also to the output layer.

**Figure 1:**
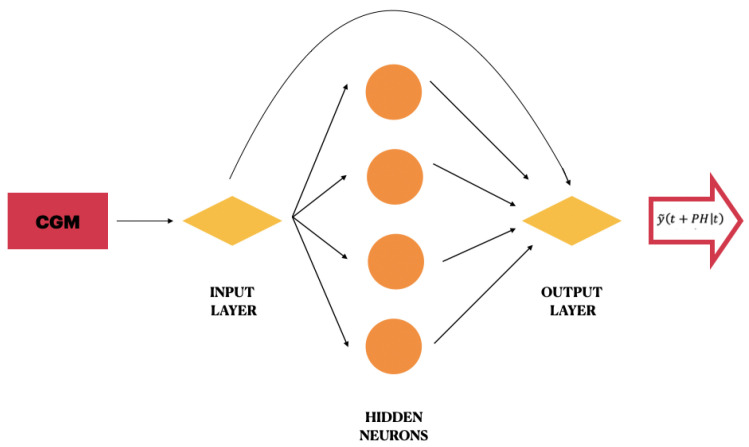
Schematic illustration of the proposed Jump Neural Network.

This model was originally proposed by Zecchin et al. [15] for predicting the blood glucose levels of 10 subjects with T1DM in daily-life conditions through exploiting a multivariate approach. The network used in this work presents only one hidden layer composed of four neurons with sigmoidal activation function. In practice, it differs from the original model of Zecchin because it has a different number of hidden neurons and exploits a univariate approach. A schematic illustration of the proposed Jump Neural Network is shown in Figure 1.

The regression task was conducted according to the Precision Medicine paradigm and with a univariate approach. At each prediction time stamp *t*, the network input consists of the most recent 10 min of CGM values. The network output is the future blood glucose value at time (t+PH). PH is the prediction horizon, i.e., how far forward in time a prediction is performed, and, in this work, it is set to be equal to 30 min. At each time stamp *t*, the Jump Neural Network predicts a signal that can be expressed as

(1) $$\tilde{y}\left(t+PH|t\right)=\mathrm{IOW}\cdot I{\left(t\right)}^{T}+\mathrm{HOW}\cdot f\left(\mathrm{IHW}\cdot I{\left(t\right)}^{T}\right)$$

where

- **I**(*t*) is a row vector with *M* elements corresponding to the CGM [t−M+1,t]. We experimentally found the optimal value for M=10.
- **IOW** is a row vector with *M* weight elements directly connecting every input to the output neuron.
- **HOW** is a row vector of *L* weights connecting every hidden neuron to the output neuron.
- **IHW** is a L×M matrix of weights connecting every input to every hidden neuron.
- *f* is the tangent-sigmoid activation function, computed element-wise on the results of $\mathrm{IHW}\cdot I{\left(t\right)}^{T}$.

The first term models the linear relationship between the target and the inputs, whereas the second term models the nonlinear relationship.

For each patient, each group of continuous days is considered as a stand-alone group of data. With regard to the network input, the CGM of each group of continuous days is used both as a vector of features and as a vector of labels. In detail, if a group of data is composed of *N* minutes of CGM, the time stamps ranging from 1 to (N−PH) are considered as features, and the time stamps ranging from (PH+1) to *N* are considered as labels. The vectors are then reorganized in such a manner that, at each time stamp *t*, the network receives as input 10 min of CGM data (i.e., the time window [(t−9),t]) that are associated with the label corresponding to the value of CGM at the time stamp (t+30).

**Figure 2:**
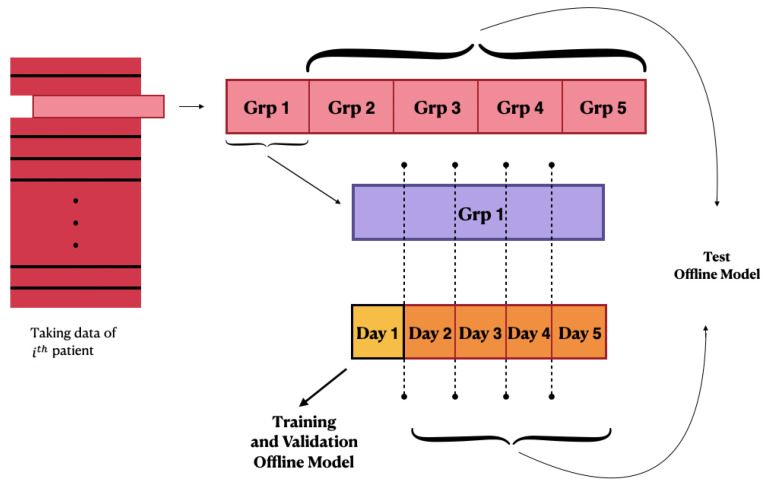
Schematic illustration of the first configuration’s behaviour.

In each of the proposed configurations, and for each patient, at the beginning, an offline training is carried out on the initial 80% of data of the first 24 h of the first group of continuous days, and the model is validated on the remaining 20% of the data. The network performance during the offline training is evaluated in terms of Mean-Squared-Error (MSE). Defining y(t) as the true CGM value at time stamp *t* and $\tilde{y}\left(t\right)$ as the related model prediction, we can define the error at a time stamp *t* as

(2) $$e\left(t\right)=y\left(t\right)-\tilde{y}\left(t\right)$$

and the MSE as

(3) $$MSE=\sum_{t=1}^{N}\frac{e{\left(t\right)}^{2}}{N}$$

where *N* is the number of time stamps of the predicted time series. After the first offline training, the process diversifies according to the configuration used:

- Offline training configuration: After training on the first available 24 h of data, all the following days are considered as test sets of the offline model, and the performance is evaluated. A schematic illustration of the first configuration behaviour is shown in Figure 2;
- Online training configuration: After the training of the offline model, the Jump Neural Network uses the online update mode. The weights obtained from the offline-trained model are used to initialize the online configuration. At each time stamp, the model makes a prediction of the blood glucose value at (t+PH). Every five timestamps, the configuration of the network is updated using the 24 h of CGM immediately preceding the test instant, and the training and test windows are moved forward by 5 min. The online training performance is evaluated in terms of the MSE. This process is iterated until all of the patient’s CGM samples have been considered as test sets, so that every prediction is performed after training the model with the most recent 24 h of data. Therefore, this configuration presents a considerably greater computational burden compared with the previous one;
- Online training configuration with penalty: the model is trained online and works similar to the second configuration; however, the performance during the online training phase is evaluated through the MSE product with a penalty. The latter was generated starting from the difference between the current value and the predicted value.

**Figure 3:**
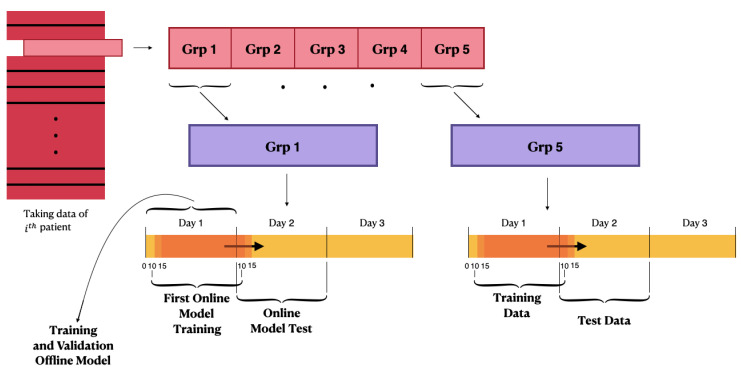
Schematic illustration of the second and third configurations’ behaviour.

A schematic representation of the latter two configurations is illustrated in Figure 3. As introduced, the third configuration is characterized by a custom loss function built *ad hoc* to introduce the characteristics of reinforcement learning into the model. The custom loss is composed of the product between the squared errors and the penalty variable:

(4) $$penalized\;loss=\sum_{t=1}^{N}\frac{e{\left(t\right)}^{2}\cdot penalty\left(t\right)}{N}$$

where the latter is defined as follows:

(5) $$penalty\left(t\right)=\left\{\begin{array}{cc} 0, & \mathrm{if}\;e(t)\leq 5\\ 1, & \mathrm{if}\;5<e(t)\leq 10\\ 2, & \mathrm{if}\;10<e(t)\leq 20\\ 0.5\cdot e(t), & \mathrm{if}\;e(t)>20 \end{array}\right.$$

The construction of the penalty is designed to make the model act more rigidly when the error is larger, trying to improve the prediction on those time stamps where the error is great while taking less into account those time stamps where the error is small.

## 3. Results and Discussion

All the simulations were performed using Google Colab and the open-source libraries of Keras and TensorFlow. In the offline training phase, a learning rate of 0.1 and a maximum number of epochs equal to 500 were set for the network training. The early stopping technique was also used, i.e., the network finished its training if the validation set had no improvement in terms of accuracy for 10 consecutive validation checks carried out every four epochs, in order to prevent overfitting. With regard to the configurations involving online training, gradient clipping was exploited to prevent the gradient explosion observed during preliminary stages, and its value was set to 0.3 from the experimental results. The learning rate was set to 0.01.

The final performance of the configurations was evaluated in terms of the Root-Mean-Squared-Error (RMSE = $\sqrt{\mathrm{MSE}}$) in order to provide an immediate comparison with other works in the literature. Different types of performance were considered for the three configurations: first, the average RMSE was calculated for each patient considering the total of days in the test set; second, the RMSE associated only to the time stamps in which each patient performed physical activity was calculated; finally, the average RMSE of all patients and for each type of physical activity was computed.

### 3.1. Offline Training Configuration

In the offline configuration, the model was trained from scratch only once for each patient, exploiting the first 24 h of data available, and tested on all the following days. Table 1 reports the results of this test for each patient. The second column reports the results referred to the total amount of recorded days, whereas the third refers only to the time stamps during which physical activity was performed. The bottom panel of the table reports the mean of the RMSEs concerning the total days and the time stamps associated with physical activity.

In detail, the two bottom lines report the average RMSE referred only the time stamps in which anaerobic or aerobic exercise was performed. Interestingly, the average RMSEs related to predictions associated with exercise were better than thsoe on the total of days. The patients for which the best performance was achieved for predictions during exercise were not the same as those that provided the best predictions on the total of days, both concerning aerobic and anaerobic exercise. The average results regarding physical activity show that the model was better at predicting the anaerobic type of activity over aerobic.

### 3.2. Online Training Configuration

In the online configuration, the model was trained from scratch only once for each patient and then updated every time new data were available. Table 2 reports the results of this test for each patient. The structure and meaning of the elements shown in the table are the same as in Table 1. Again, the results concerning physical activity were better than those concerning the total amount of days. The average results were similar to those achieved using the offline configuration. The results regarding physical activity show that this model was also better at predicting anaerobic activity over aerobic.

**Table 1:** Results of the tests of the offline configuration. The results are reported in terms of average RMSE (mg/dL). For each patient, we report whether they performed aerobic (AE) or anaerobic (AN) exercise. The second column reports the results referring to the total amount of recorded days, whereas the third refers only to the time stamps during which physical activity (PA) was performed. The average RMSEs are also reported. The bottom panel reports the average RMSE related only to anaerobic and aerobic exercise.

Patient ID	RMSE Total Days	RMSE PA	PA Type
Patient 1	22.0	23.2	AN
Patient 2	20.7	21.6	AN
Patient 3	25.1	17.8	AN
Patient 4	22.8	29.6	AE
Patient 5	29.0	23.7	AE
Patient 6	29.9	25.6	AE
Average RMSE	24.9	23.5	-
Average RMSE—AN	-	20.8	-
Average RMSE—AE	-	26.3	-

### 3.3. Online Training Configuration with Penalty

In the online configuration with penalty, the model was trained from scratch only once for each patient and then was updated every time new data were available, while considering a penalty that was greater as the forecast error increased. Table 3 reports the results of this test for each patient. The structure and meaning of the elements shown in the table are the same as in Table 1. The results concerning physical activity were better than those concerning the total amount of days, and, once again, the model was better at predicting the anaerobic type of activity over aerobic. The average results were similar to those achieved using the previous configurations.

### 3.4. Comparison between the Three Configurations and the State of the Art

Table 4 summarizes the results achieved with the three configurations, together with the average results on the total days and on aerobic and anaerobic exercise. All the configurations achieved similar results, both concerning the total days and the physical activity. All the models performed better when predicting glucose levels related to anaerobic exercise rather than aerobic; this may be a sign that the abrupt glycaemia decreases that occur during aerobic physical activity are particularly difficult to predict accurately.

During aerobic exercise, glycaemic variation can be influenced by the intensity and duration of exercise, insulin to glucagon ratio, fitness, nutrition, and initial glucose concentration [24]; moreover, most patients consume snacks immediately before aerobic exercise with the aim of preventing hypoglycaemic events, and this causes an increase in glucose levels followed by a decrease due to exercise [25]. A multivariate approach taking into account such heterogeneous features may improve glucose predictions during aerobic physical activity.

Although the offline configuration achieved better results on the total number of days for only one out of the six patients compared to the online configuration without penalty, it remains the best choice for physical activity. Indeed, the offline configuration achieved better average performance for both aerobic and anaerobic physical activity. On the other hand, the offline model was outperformed by the other configurations with respect to the performance on the total days. The fact that better results of the configurations with online training on the total number of days did not translate into better results concerning only physical activity could indicate that these configurations have a better long-term adaptation.

However, the performance improvement is not great enough to justify the significant increase in the computational burden introduced by the online configurations; thus, the offline configuration would likely be most appropriate for use in a real-life application. With regard to the online configurations, the performance of the penalty and non-penalty configurations were very similar, indicating that the negative reinforcement did not present an advantage. With regard to the individual performance on the total number of days, we observed that some individual differences may affect the prediction of the three models.

**Figure 4:**
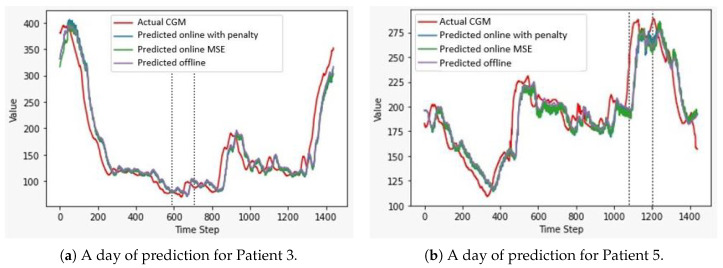
Comparison between the predictions of the three configurations on two sample days. We report the actual CGM track (red line), the predictions of the online configuration with penalty (blue line), the predictions of the online configuration trained using the Mean-Squared-Error as a loss function (green line), and the predictions of the offline model (purple line). All the predictions are almost overlapped and slightly shifted from the original CGM values. The black dotted lines delimit the period during which physical activity was performed. (**a**) Predictions on a whole day of Patient 3, who performed anaerobic exercise (RMSEs between 24.2 and 26.0 mg/dL). (**b**) Predictions on a whole day of Patient 5, who performed aerobic exercise (RMSEs between 27.8 and 29.7 mg/dL).

The configuration with online training achieved the best performance for Patients 2, 3, and 6, whereas the online and offline training configuration equally achieved the best performance for Patient 1, and the online configuration with penalty achieved the best performance for Patients 4 and 5. As no clear pattern was observed using one configuration with respect to another, we must conclude that the small variations of performance may be due to individual differences between patients. A similar analysis can be applied to the timestamps related to PA. Figure 4 shows a graphical comparison of the predictions of the three configurations on a sample day of Patient 3, who performed anaerobic physical activity, and Patient 5, who performed aerobic exercise; the time stamps in which physical activity was performed are also highlighted.

As mentioned in the Introduction, the forecasting of the blood glucose levels of T1DM patients that perform physical activity has rarely been addressed in the literature mainly due to the difficulties in collecting data. Despite the reduced amount of published works, a partial comparison can be performed with the work of Hobbs et al. [17], which is likely the most remarkable work resorting to a regression task. They achieved an average RMSE of 29 mg/dL on 32 T1DM subjects who practised skiing and snowboarding, which are considered to be mainly anaerobic types of exercise.

All the configurations proposed in this work achieved considerably better performance on anaerobic physical activity, although the number of patients investigated is exiguous. Furthermore, differently from other works in the literature, the proposed model exploits only the previous knowledge of CGM data without requesting the patients to manually provide information on their status [16,20] or integrating data from several heterogeneous sensors [17], making it a promising approach for future developments. However, we acknowledge that the results achieved cannot be conclusive nor exhaustive because they were achieved on a small number of patients, and therefore they need to be validated on a larger dataset in order to be considered definitive.

## 4. Conclusions

This work aimed to implement a blood glucose level forecasting model and to validate this model during physical activity on subjects suffering from T1DM. A comparative analysis was conducted between the different training configurations developed. A Jump Neural Network was used to forecast the blood glucose levels of six adults with diabetes during physical activity. Regarding the methods of application of the algorithm, three different configurations were developed, which included a configuration with offline training, a configuration with online training, and a configuration with online training and a loss function that incorporated a negative reinforcement in the case of great differences between the predicted and real values.

A comparative analysis was then conducted, with the aim of determining whether the performance of a configuration with offline training could be exceeded by configurations with online updates, with and without penalty contribution. The online configurations performed better than the offline configuration in total days but not on the data related only to the CGM associated with the physical activity, which could indicate that these configurations have a better long-term adaptation. Even though the average results of the online configurations outperformed those of the offline configuration in the total days, the results do not justify the increased computational burden because the improvement was not significant.

Future developments could be addressed regarding the study of negative reinforcement—this time associated with the offline model—which could be different and more functional in terms of setting its value with respect to the difference between the true and predicted values, and of the method of insertion in the loss function. It would be interesting to collect data from patients who perform both AE and AN exercise, in order to assess with more confidence whether the better results obtained for AN exercise are due to the type of physical activity rather than to inter-patient variables. In addition, people with T1DM who regularly practice sports report that they follow a routine of insulin adjustments and carbohydrate ingestion before and after the exercise, which may represent additional features along with heart rate.

## Figures and Tables

**Table 2:** Results of the test of the online configuration. The results of performed tests are reported in terms of the average RMSE (mg/dL). For each patient, we report whether they performed aerobic (AE) or anaerobic (AN) exercise. The second column reports the results referred to the total amount of recorded days, whereas the third refers only to the time stamps during which physical activity (PA) was performed. The average RMSEs are also reported. The bottom panel reports the average RMSE related only to anaerobic and aerobic exercise.

Patient ID	RMSE Total Days	RMSE PA	PA Type
Patient 1	22.0	23.3	AN
Patient 2	20.1	21.0	AN
Patient 3	24.2	18.9	AN
Patient 4	22.8	30.2	AE
Patient 5	29.7	24.7	AE
Patient 6	28.3	25.2	AE
Average RMSE	24.5	23.9	-
Average RMSE—AN	-	21.1	-
Average RMSE—AE	-	26.7	-

**Table 3:** Results of the test of the online configuration with penalty. The results of performed tests are reported in terms of the average RMSE (mg/dL). For each patient, we report whether they performed aerobic (AE) or anaerobic (AN) exercise. The second column reports the results referred to the total amount of recorded days, whereas the third refers only to the time stamps during which physical activity (PA) was performed. The average RMSEs are also reported. The bottom panel reports the average RMSE related only to anaerobic and aerobic exercise.

Patient ID	RMSE Total Days	RMSE PA	PA Type
Patient 1	23.0	24.2	AN
Patient 2	21.1	22.2	AN
Patient 3	26.0	17.3	AN
Patient 4	21.7	28.7	AE
Patient 5	27.8	22.4	AE
Patient 6	28.5	28.5	AE
Average RMSE	24.6	23.9	-
Average RMSE—AN	-	21.2	-
Average RMSE—AE	-	26.5	-

**Table 4:** Results of the test of all three configurations. The results of the performed tests are reported in terms of the average RMSE (mg/dL). For each patient, we report whether they performed aerobic (AE) or anaerobic (AN) exercise. The results were calculated both for the total number of days and only for the CGM forecasts actually associated with physical activity (PA). For the latter, in addition to the total average, the average of the RMSEs associated only with anaerobic and aerobic PA is also reported.

Patient ID and PA Type	Total Days Offline	Total Days Online	Total Days Online Penalty	PA Offline	PA Online	PA Online Penalty
Patient 1—AN	22.0	22.0	23.0	23.2	23.3	24.2
Patient 2—AN	20.7	20.1	21.1	21.6	21.0	22.2
Patient 3—AN	25.1	24.2	26.0	17.8	18.9	17.3
Patient 4—AE	22.8	22.8	21.7	29.6	30.2	28.7
Patient 5—AE	29.0	29.7	27.8	23.7	24.7	22.4
Patient 6—AE	29.9	28.3	28.5	25.6	25.2	28.5
Average RMSE	24.9	24.5	24.6	23.5	23.9	23.9
Average RMSE—AN	-	-	-	20.8	21.1	21.2
Average RMSE—AE	-	-	-	26.3	26.7	26.5

## Data Availability

The data presented in this study are available on request from the corresponding author. The data are not publicly available due to privacy.
